# Conflict, Crisis, and Abuse in Dharavi, Mumbai: Experiences from Six Years at a Centre for Vulnerable Women and Children

**DOI:** 10.1371/journal.pmed.1000088

**Published:** 2009-07-07

**Authors:** Nayreen Daruwalla, Armida Fernandez, Jenny Salam, Nikhat Shaikh, David Osrin

**Affiliations:** 1 Society for Nutrition, Education and Health Action, Urban Health Centre, Chota Sion Hospital, Mumbai, Maharashtra, India; 2 UCL Centre for International Health and Development, Institute of Child Health, London, United Kingdom

## Abstract

Nayreen Daruwalla and colleagues describe the Centre for Vulnerable Women and Children, which serves clients coping with crisis and violence in the urban setting of Dharavi, Mumbai.

**Summary Points**

- Violence against women is common in India, but service provision to address it is limited.
- The Centre for Vulnerable Women and Children serves clients coping with crisis and violence in the challenging urban setting of Dharavi, Mumbai.
- We discuss factors that shaped the development of the Centre over six years.
- Intervention was often guided by clients' desire to keep their families together.
- Successful intervention requires strong links with health care providers, the police, legal services, and community-based organisations.

## Context

India's latest National Family Health Survey reports violence against 37% of ever-married women [1]. This has now been recognised as a serious problem, but most reports have concentrated on quantifying its burden. Despite some work on rehabilitation [2],[3], and some evidence that advocacy and counselling services are effective, accounts of experience have been limited [4],[5]. The Centre for Vulnerable Women and Children has been running for six years in Dharavi, Mumbai, and we have recently reviewed our records and experiences in order to plan an expansion of activities. We took the opportunity to reflect on the challenges of developing and sustaining a crisis intervention centre in urban India.

The Centre opened in late 2000 in response to the experiences of health workers at Lokmanya Tilak Municipal General (LTMG), Mumbai's largest public hospital. Many victims of domestic violence came to the hospital, but interaction with doctors and nurses tended to stop at treatment for injuries [6]. Engaging with the wider issues—emotional, psychiatric, social, and legal—requires confidence, time, training, protocols, and resources, all of which are in short supply. The Centre was conceived as a means to address this gap through a partnership between the Municipal Corporation and a non-government organisation (NGO). Founded at a municipal health facility in Dharavi, one of the world's largest urban slum areas, the Centre was located within the LTMG client community (a plus point), but not within LTMG Hospital. One could argue that this relieved the hospital of pressure to engage with the issue of domestic violence, or that flexibility in NGO–government collaborations allows piecemeal progress.

**Figure 1:**
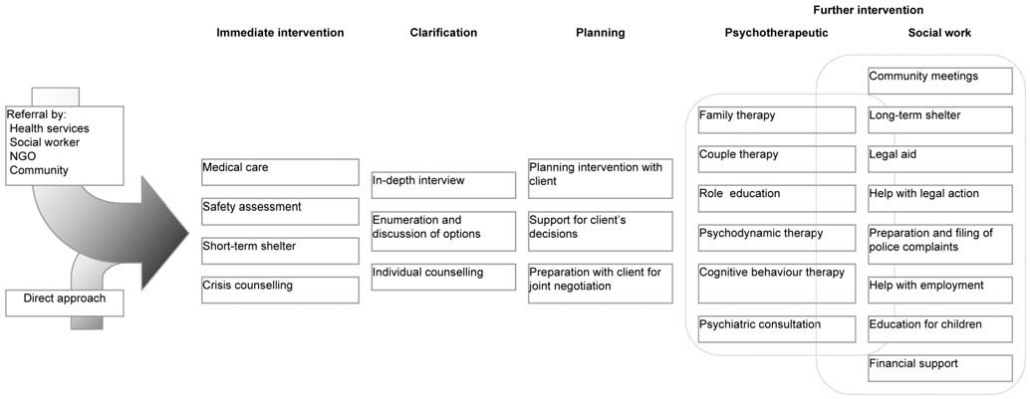
Centre for Vulnerable Women and Children intervention model.

Figure 1 summarises the Centre's activities, which include both psychotherapy and social work. We act quickly, arrange medical care and temporary shelter if necessary, provide immediate and longer-term counselling for the client and her family, and facilitate intervention at a range of levels. We deal with both violence and crisis. Gender-based violence is any act “…that results in, or is likely to result in, physical, sexual or psychological harm or suffering to women, including threats of such acts, coercion or arbitrary deprivation of liberty, whether occurring in public or in private life” [7]. India's recent Protection of Women from Domestic Violence Act (2005) provides a more detailed definition in a similar spirit [8]. Crisis develops from a change in circumstances that disrupts individual and family patterns of function, with limited ability for resolution through usual approaches; and intervention involves support for psychosocial functioning during the period of disequilibrium. We take a client-centred, non-directive approach based on the humanistic therapy of Carl Rogers [9]. This common approach emphasises actualisation and the development of one's potential. We try to help clients to understand their experiences, acknowledge the choices available, and take responsibility for their actions to deal with present realities [10]. In encouraging our clients to trust themselves to manage the present, we hope that their ability to solve problems in the future will increase.

## Reasons for Seeking Help

The Centre's activities have been shaped by the reasons that people consult us, and by the trade-off between what they want us to do, what we would like to do, and what we can do. Seven hundred and fifteen clients sought help from 2001 to 2006, the numbers increasing annually. Most were women in abusive situations on a background of conflict with their partners or families (Tables 1 and 2). Relationships were further coloured by disputes about property, earnings, or dowry [11],[12],[13], accusations of infidelity by clients or their partners, and the stresses of multiple marriages.

**Table 1:** Issues underlying consultation, for 661 adult female clients, 2001–2006.

Issues	Frequency	%
Abuse	604	91
Difficulties in relationship with partner	446	64
Difficulties in relationship with family	243	35
Conflict or worry about client offspring or the partners of their offspring	29	4
Financial, property, and dowry conflicts	216	31
Addiction in client or partner	169	24
Illness in client, partner, or offspring	146	21
HIV-related concerns	17	2
Client split from partner or family: desertion, eviction	107	15
Accusation of client or partner infidelity	92	13
Problems in marriages with multiple wives	42	6
Bereavement	41	6
Death of partner	23	3
Family difficulties in accepting relationship	16	2
Custody and paternity disagreements	12	2
Fertility or son preference issues	9	1
Unmarried pregnancy	7	1
Caste issues	6	<1
Child abuse	5	<1

Categories are not mutually exclusive since clients often identified more than one problem.

**Table 2:** Abuse reported by 661 adult female clients, 2001–2006.

Characteristics of Abuse	Frequency	%
Any abuse^a^	604	91
Emotional abuse	601	91
Physical abuse	532	81
Sexual abuse	239	36
Economic abuse	166	25
**Primary perpetrator of abuse (** ***n*** ** = 471)**		
Partner	342	73
In-laws	79	17
Natal family members	21	5
Other relatives or community members	15	3
Offspring or their partners	12	3
**Frequency of abuse (** ***n*** ** = 510)**		
Ongoing	467	92
Occasional	23	5
Single event	20	4
**Significant injury associated with physical abuse (** ***n*** ** = 471)**	**96**	**20**
**Means of violence (** ***n*** ** = 95, not mutually exclusive)**		
Hit, kicked, punched, pushed, hair pulled	35	45
Hit with object	27	35
Accidental injury (client's injury reported as accidental, even though associated with abuse)	6	8
Burned	7	7
Doused with kerosene	4	5
Cut or pierced with blade or glass	3	4
Jumped from height or train (because of stress resulting from abuse)	2	3
**Injury (** ***n*** ** = 90)**		
Bruising, cuts, bites	55	61
Head injury	10	11
Fracture or joint trauma	8	9
Burns	7	8
Bleeding, internal or external	6	7
Foetal loss, pregnancy complications	3	3
Poisoning	1	1

a We define abuse as emotional, physical, sexual [1], or economic; assuming that sexual abuse implies physical abuse, and that both imply emotional abuse.

## Challenges and Responses

Reviewing our experiences, we think that three issues particularly influenced the Centre's development: the relative invisibility of the problems with which we are trying to deal; women's desire to meet normative expectations and to keep the family together; and a spiralling need to connect with other service providers, families, and communities.

Violence against women is common and tolerated. It is difficult to elicit reports of it [14], and only a small proportion of women seek help [11],[12], usually from family, friends, and neighbours [1],[11],[15]. Although daily abuse is common [16], the likelihood of consultation and reporting increases with the severity of violence and the need for care for injuries [13],[17]. Building community involvement is, therefore, challenging. Women's subordinate role in the family, including the notion that transgression may invite appropriate punishment, is hard to address. When consultations increased in the first years of operation, referrals tended to be vague and clients asked for financial help rather than other support; in a precarious position, financial security is an appropriate priority. Our service-based viewpoint also made it difficult to estimate the prevalence of abuse and violence and to ascertain the outcomes of our involvement. To a degree, we had to accept this limitation. Clients came to us in need, and once their immediate concerns were addressed they usually found their own mechanisms for dealing with their problems. If our aim was to help them become independent and resilient, and to argue for their rights, we hope that a lack of sustained contact may reflect success. It may equally be a circular means of justification.

The ubiquity of abuse and the consequences of familial disruption affect women's aspirations and put a ceiling on hope. Finances and family honour are disincentives to separation, and most women remain with their partners, saying that violence is normal in a marriage [11]. A recurring theme is a perceived failure to live up to expected family roles and responsibilities. In most cases the failure is on the part of the client (as a wife, mother, daughter-in-law, or wage-earner), particularly if she has married young and been unable to adapt to the new family setting (see Box 1) [6],[12]. Violence is often a mechanism for enforcing family expectations [18], its occurrence and toleration modified by socio-cultural norms [15],[19]. Lapses in fulfilling perceived responsibilities—cooking, household chores, looking after children and in-laws—are often used to justify abuse [11],[20], as is disobedience to one's husband [15].

**Box 1. Vignette: Shraddha**

Shraddha* was referred by one of the Centre's field staff. She had a black eye and facial bruising. Her husband had beaten her up because she did not obey his dictate not to go out on her own. She was only allowed to go to her grandmother's house, and her elderly grandmother accompanied her to the Centre. Shraddha had been married off early due to financial pressures on her family. Her parents had died, and at the age of 20 she was grappling with her marital problems and nursing a 15-month-old baby. When we met her she was crying continuously and said she felt helpless. Nevertheless, she asked us to assist in reconciliation with her husband and said that she wanted to stay with him. Before accessing the Centre she had approached the police and had twice registered minor offences without effect.

Our caseworkers helped Shraddha to get medical care and provided crisis counselling. We planned further intervention and visited her husband at home to ask him to come to the Centre. Shraddha's husband complained that she was unable to perform her duties as a wife. She failed to cook food and take care of her child, and he was suspicious of her activities. He did not allow her to dress well and criticised her constantly. Initially, he did not take the counselling sessions seriously, but responded to some pressure to do so when we involved the police. Over a lengthy period of mutual role education and marital therapy, the abuse stopped.

Today Shraddha is a mother of two children. She lives happily with her husband and the two of them regularly attend programmes at the Centre. Shraddha was determined to retain her own identity, and has taken up a number of training programmes and become a peer educator. She helps other women facing violence and has become an important link between the community and the Centre. She often talks of her aspirations: “I feel that my life has completely changed. Once upon a time my husband used to beat me and not allow me to go out of the house, and today he encourages me to help other women. The Centre has helped my self-respect and dignity and I have become like a bird with graceful wings.”

* The clients in this manuscript have given written informed consent as per PLoS consent policy to publication of their case details. Their names have been changed.

There is a premium on keeping families intact, avoiding parental shame and the emotional, social, and financial consequences of separation. We need to understand this. Almost all our clients receive individual counselling (97%), but it rapidly became clear that they wanted us to negotiate on their behalf with partners and family members. We became convinced that this was crucial, particularly since it seemed more productive to try to change the attitudes of perpetrators of violence. Helpfully, the idea appealed to parents, in-laws, and existing community bodies, and we steadily expanded the role of partner, family, and community counselling.

The need to build networks, and the question of whom they should include, underpins current activities. Links with health workers catalysed the Centre's foundation, but it became clear that they had to be strengthened. Women's health often suffers as a result of early marriage [20], pregnancy, and exploitation for housework. Women who report good health are less likely to report abuse [11], and women who have experienced violence are more likely to report poor health [6],[21],[22]. Perhaps only half of those who need health care in this context receive it [11]. Clients reported significant injury in one-fifth of situations involving physical abuse, and 14% required help with accessing medical services for either injury or mental illness. Illnesses were also sources of both financial strain and family stress, a finding that led us to consolidate links with psychiatrists. Clear lines of communication are mutually beneficial: we need access to health care, and doctors benefit from knowing where they can refer clients in crisis.

Despite our connections with municipal health services, shelter (required in 10% of cases) remains a problem. We have negotiated the allocation of ten beds in a medical ward of the Dharavi health centre, but women with injuries or mental illness are not admitted and medico-legal procedures are required. The relatively insecure environment has meant that perpetrators of abuse have had access to women who have been admitted for their protection. We have also found it difficult to broker solutions for couples dealing with alcohol dependency (addictions were common, over 90% of them reported in partners, and often led to disputes over earnings and the allocation of household budgets), and referrals for reduction management have not been successful. This has been particularly disappointing because awareness activities organised in collaboration with the police and other NGOs have led to an increase in approaches from women dealing with partner dependency.

Connections with police and legal services must be strong. A third of clients need help preparing legal and police documents, and visits to police stations and courts constitute a substantial part of casework. Clients' awareness of their legal rights is generally limited and action is further constrained by their poverty. This was also true for us; initially, we were neither knowledgeable nor credible sources of advice on legal process. We have improved with experience, but legal support remains problematic. At one stage we offered free legal counselling, but subsequent intervention incurred a cost. This led to complaints by clients who had understood that it would be free, and after negotiation the Municipal Corporation initiated a free legal aid cell. We have probably been more successful in developing links with the police. The Domestic Violence Act enumerates the duties of public servants at a number of levels, but the police generally find it easier to intervene in cases of acute physical violence than in ongoing domestic abuse. We have organised sensitisation workshops and health consultations at local police stations, which have strengthened the partnership, triggered more referrals, and led to the organisation of local awareness events.

**Figure 2:**
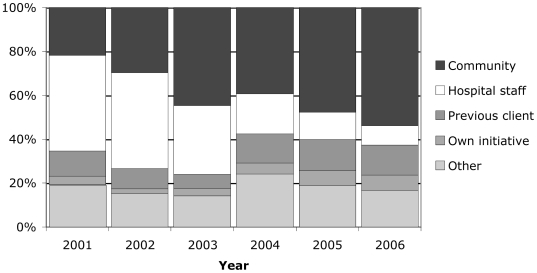
Routes by which women in crisis approached the Centre, 2001–2006.

The most significant development over the last six years has been a gradual shift in emphasis from institutional support to community action. The pattern of referral has changed over time, reflecting a move from health service to community sources (Figure 2), and we have worked steadily more with other NGOs to organise sensitisation workshops and advocacy meetings. Because issues like employment and income generation are open doors for community intervention, while domestic violence is not, we have developed an NGO forum in which violence against women is just one of a number of issues. We have organised awareness campaigns, theatre performances, and gender-sensitive competitions. We have also adopted a micro-planning process in which community groups identify their problems, recognise local resources, prioritise the area of intervention, and build plausible action plans.

One effect has been that community members have themselves become catalysts for change, with concerned individuals helping out women in crisis. The first contact for a woman in crisis is often a close confidant, and in seeking feasible solutions we have learned that the suggestions of friends and neighbours are often the most practical and acceptable. This extends to advice from community associations (*samaj*) and women's groups (*mahila mandals*), whose inputs have been particularly useful in achieving settlements for marital discord. We have helped to form 45 local women's groups to provide initial support and crisis intervention, and four local committees who work on initiatives to put the reduction of domestic violence on the public agenda.

This community expansion has also relieved our four caseworkers. Their work is inherently stressful, there are often conflicting versions of events (see Box 2), and the effects of interventions appear slowly. When they are unsuccessful, caseworkers may be blamed by clients or community members; this happened after the murder of a pregnant woman who had approached the Centre for help, but had not permitted us to negotiate with family members because of fears that they might expel her. This raises the question of what we mean by collaboration. If we are working with communities in which violence is the norm, the extent to which the Centre should be involved in activism for social justice remains uncertain. When a young woman died from burns, our partnership with the Municipal Corporation (and the potential media exposure) was a cause for anxiety. We decided to attend community meetings and use our existing relationships to facilitate access to police and legal services. The case also catalysed the organisation of local meetings to discuss the issue. When a second young woman was murdered by her husband, community members approached us for support. We were able to help with a litigation process that led to life sentences for the perpetrators. Small milestones lead to trust.

**Box 2. Vignette: Nagamma**

Nagamma*, aged 30, was referred by a children's shelter. She had asked the organisation for shelter for her two children, a boy and a girl, as her partner had an alcohol problem and did not take care of them. Nagamma was in an abusive relationship in which she and her partner were not married and he would leave and return periodically. He had recently beat her severely, leaving her immobile and in pain, before leaving again. At this point, Nagamma had decided to separate. Her partner continued to visit their children at school and had harassed teachers if they did not grant him access. Nagamma's decision was to institutionalise the children until they reached majority. In the meantime she would work to earn a living and save for the children's future.

Caseworkers from the Centre made visits to the children's school and Nagamma's partner's home, where staff and her partner's sister corroborated her account. With this as support, we put a case for shelter to the organisation that had originally referred them. This application was unsuccessful, and we presented the case to a child welfare committee, which arranged shelter at a government children's home. In the interim, Nagamma's partner had found out about the shelter arrangement and had begun to harass the superintendent of the children's home. The superintendent referred him to the Centre, and we engaged in a series of counselling sessions. He took the position that he wanted custody of his son, but not his daughter. Nagamma was not willing to hand over either child, and the Centre supported her decision and suggested that her partner file for legal custody. We also helped her to prepare her own legal submission. The children were moved once again to another shelter, where Nagamma was able to live with them. At present, she is working at the shelter and her children are going to school.

* The clients in this manuscript have given written informed consent as per PLoS consent policy to publication of their case details. Their names have been changed.

## Next Steps

The strongest message from our work is that the ramifications of violence and crisis are so complex that a stand-alone service will under-perform. A crisis centre requires community involvement and strategic alliances with parallel systems. We have short-term objectives—strengthening legal and police links, finding a secure and stable source of shelter—but also a more ambitious agenda to advocate citywide recognition of violence against women and children as a public concern. Sustainability will depend on partnerships. Along with legal resource strengthening, we aim to help set up a crisis cell within LTMG Hospital, and to pilot a model of alliances between NGOs, community-based organisations, and community members in Dharavi.

## Abbreviations

LTMG: Lokmanya Tilak Municipal General
NGO: non-government organisation
